# *Helicobacter pylori* Infection Maybe a Risk Factor for Cardiac Syndrome X

**DOI:** 10.3389/fcvm.2022.823885

**Published:** 2022-07-15

**Authors:** Dong-Hong Zhang, Chen Yuan, Bei-Bei Wang, Xin-Jiang Dong, Shu-Ping Lv, Fei-Hong Li, Zhen-Xiu Hou, Xiao-Li Liu, Kang Chen

**Affiliations:** ^1^Department of Infection Disease, Shanghai Fifth People's Hospital, Shanghai, China; ^2^Department of Emergency, Shanghai Fengxian District Central Hospital, Shanghai Jiao Tong University Affiliated Sixth People's Hospital South Campus, Shanghai, China; ^3^Department of Cardiology, The First People's Hospital of Jinzhong, Jinzhong, China; ^4^Department of Cardiology, Shanxi Cardiovascular Hospital, Taiyuan, China; ^5^Department of Cardiology, Shanxi Bethune Hospital, Shanxi Academy of Medical Sciences, Taiyuan, China; ^6^Department of Cardiology, Yantai Yeda Hospital, Yantai, China; ^7^Department of Cardiology, Yun Cheng Central Hospital, Yuncheng, China

**Keywords:** *Helicobacter pylori* infection, Cardiac syndrome X, cytotoxin-associated gene A, meta-analysis, systematic review

## Abstract

**Purpose:**

Cardiac syndrome X (CSX) is a condition with normal coronary angiography but angina pectoris. Chronic inflammation caused by *Helicobacter pylori* (*H. pylori*) infection may play a pathogenic role in CSX. Therefore, we conducted a meta-analysis to explore the relationship between *H. pylori* infection and risk of CSX.

**Methods:**

A systematic search in the Web of Science, Medline, Embase and Chinese databases (CNKI and Wanfang) was conducted up to October 2021. Articles on the association between *H. pylori* infection and the risk of CSX were included and were analyzed by R software (version 4.1.0).

**Results:**

Ten case-control studies involving 703 CSX patients and 731 healthy controls were included. *H. pylori* infection was associated with an increased risk of CSX (OR: 8.29, 95% CI: 4.64–14.82). We also found a significant association in those 25–40 years of age (OR: 1.34, 95% CI: 1.04–1.72), those 40–50 years of age (OR: 11.27, 95% CI: 4.29–29.61), those over 50 years of age (OR: 7.18, 95% CI: 3.59–14.36), those in developing countries [Iran (OR: 12.99, 95% CI: 8.61–19.60) and China (OR: 5.14, 95% CI: 3.09–8.56)]. However, this association was not apparent in a developed country [Italy (OR: 0.93, 95% CI: 0.37–2.33)].

**Conclusions:**

Our study suggested a possible association between *H. pylori* infection and the risk of CSX. Its pathogenicity is stronger in middle-aged individuals and some developing countries. However, more studies are needed to further investigate whether early eradication of *H. pylori* can reduce the incidence rate of CSX, especially in middle-aged individuals and some developing countries.

## Introduction

*Helicobacter pylori* (*H. pylori*) is a gram-negative bacterium that causes a variety of gastrointestinal disorders, including chronic gastritis, duodenal ulcer and gastric cancer (1, 2). It may also cause some extra-intestinal diseases, some of which are manifested as respiratory diseases and functional ischemic heart disease (1, 2), recently found to be related to Cardiac Syndrome X (CSX) (3–5). Virulent strains *H. pylori* can be divided into two subgroups based upon the expression of (6) an immunodominant, 120–145 kDa protein, cytotoxin-associated gene A (CagA). Infection with CagA may be a strong risk factor for the development of CSX by inducing endothelial dysfunction (7).

CSX is characterized by typical exertional angina pectoris, positive electrocardiograph (ECG) or exercise treadmill test, normal coronary angiography with the exclusion of coronary artery spasm. In angina patients undergoing angiography, up to 20% of patients have this symptom (3). Despite extensive studies, the mechanism of this syndrome is still unclear. Nevertheless, it has been proposed that coronary artery endothelial dysfunction is the main pathogenic mechanism underlying CSX (5).

While many studies have attempted to reveal the role of *H. pylori* infection in CSX, their results are contradictory. Thus, we conducted a meta-analysis of relevant articles in an attempt to reveal the association between *H. pylori* infection and the risk of CSX.

## Materials and Methods

Our meta-analysis strictly adhered to the Preferred Reporting Items for Systematic Reviews and Meta-Analyses (PRISMA) (8).

### Article Search

The meta-analysis was performed with a structured articles search, using the Web of Science, Medline, Embase and Chinese databases (CNKI and Wanfang). The keywords “*H. pylori*” or “*Helicobacter pylori*,” combined with “Cardiac syndrome X,” “microvascular angina,” “CSX,” and “MVA” were used as search terms. There were no language restrictions in the search. The references in the identified articles were checked and if suitable, were also included in the search. The deadline for searching was October 2021.

### Article Selection

We repeated all selections. The final inclusion of articles was decided upon by consensus, and when this decision failed, the third author (LXL) made a ruling.

Observational studies (cohort, case–control and cross-sectional) were included if the following criteria were met:

1. Studies were conducted in humans.
2. *H. pylori* infection was determined by serological tests, including antigen-specific enzyme-linked immunosorbent assay (ELISA) and Western blotting, or non-serological tests including rapid urease test, and ^13^C-urea breath test (UBT) according to the manufacturer's instructions.
3. The diagnosis of CSX included the following criteria: typical history of angina and normal coronary angiography; atypical chest pain with an abnormal myocardial perfusion Image; or exercise electrocardiogram and completely normal results on coronary angiography, with no inducible spasm on ergonovine-provocation test.
4. A control group was included. Studies with matched and sufficient information on the association between *H. pylori* infection and the risk of CSX were selected.
5. For articles with data published more than once, only the article with an adequate study strategies and large number of cases was selected.
6. Patients with evidence of left or right ventricular dysfunction, cardiomyopathy, valvular heart disease, myocardial infarction, concomitant acute and chronic disease were excluded.

### Data Extraction

Two well-trained researchers (LSP and LFH) independently extracted the following data based on the pre-specified scheme: first author or second author; average age of case group; year of publication; country; study size; case type; control type; and matching variables. All data were double input.

### Data Analysis

Our meta-analysis was conducted according to the recommendations of Cochrane Collaboration and meta-analysis report quality guidelines (9, 10) and was performed using R software (version 4.1.0). The *I*^2^ statistics were used to assess statistical heterogeneity. A value >50% was considered to have significant heterogeneity and random effect models were used to pool data. Otherwise, the fixed effects model was used. The estimated effect measure was the odds ratio (OR) with 95% confidence intervals (95% CIs) for the dichotomous data. If the 95% CI did not contain the value one, the OR was considered to be statistically significant.

The Egger's and Begg's tests were used to assess potential publication bias. Each article in this analysis was sequentially deleted to determine how much it contributed to the overall effect size. The Newcastle-Ottawa Scale (NOS) was adopted to score all articles. Articles that scored ≥7 points were considered “high-quality articles.” Several subgroup meta-analyses were performed based on country, mean age, case type, *H. pylori* detection method, socioeconomic status and year of publication.

## Results

### Search Result

**Figure 1:**
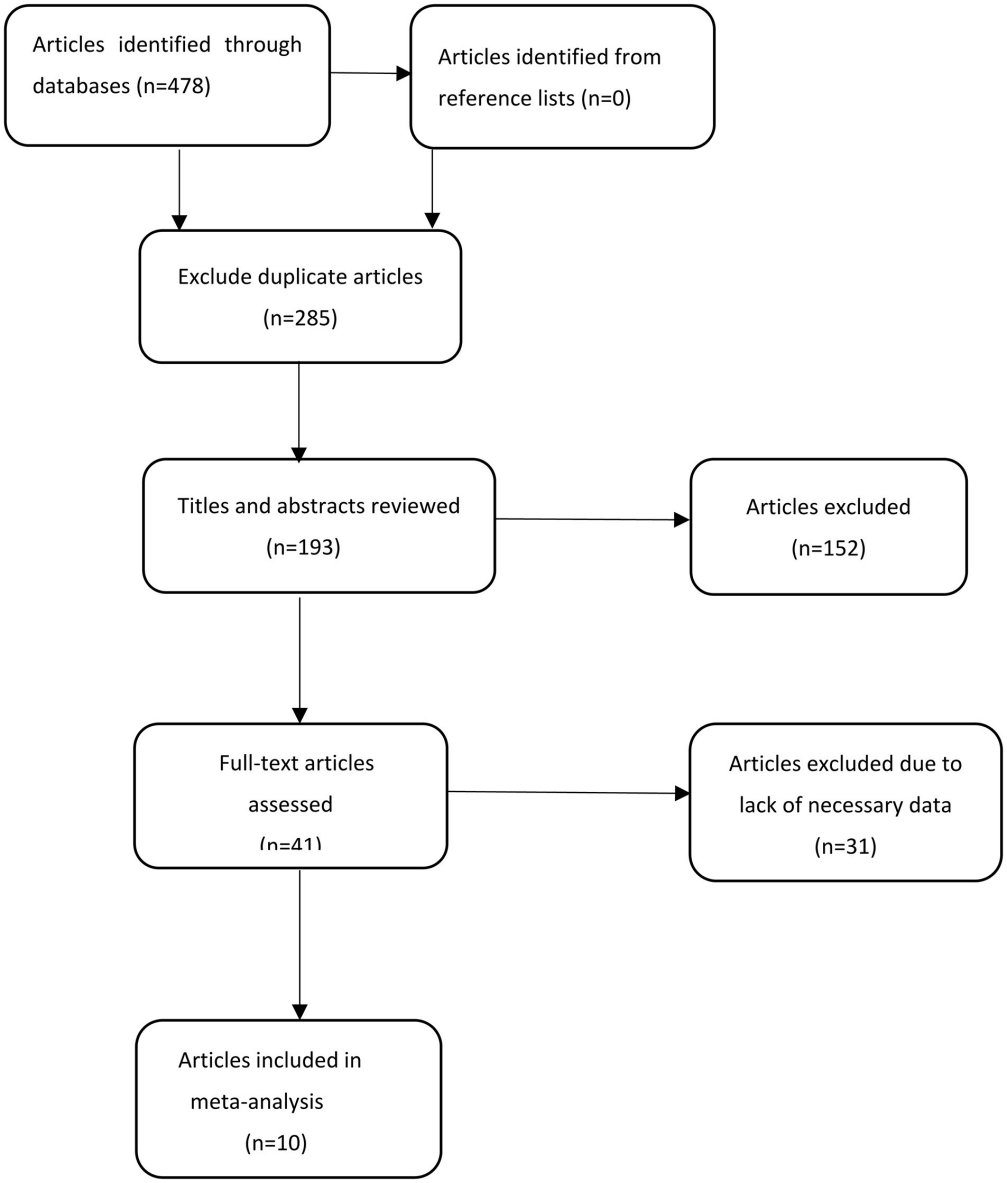
Selection process for articles included in the meta-analysis.

Using our keywords and the references from identified articles, a total of 478 potentially relevant articles were identified. After excluding duplicates, 193 articles remained, of which 152 articles were excluded after checking the title and abstract. After carefully assessing the full text of the remaining 41 articles, 31 articles were determined to not meet inclusion criteria. This was because: (1) They were comments/reviews/case reports without raw data; or (2) They were other special studies, such as *in vitro* studies, animal studies, or epidemic pathological studies focusing on the relationship between *H. pylori* infection and coronary artery disease, rather than CSX. After this exclusion, 10 articles remained in our study (3, 11–19). The selection process for this meta-analysis is shown in Figure 1. The detailed information of all identified articles is shown in Table 1.

**Table 1:** Characteristics of studies in the meta-analysis.

**References**	**Year**	**Country**	**Mean age ^**a**^**	**Study size CSX/CG**	**Case type**	**Control type**	**Study type**	**Agent**	**Matched variables^**b**^**	**Quality score**
Eskandarian (3)	2006	Iran	45 ± 5	40/40	Community	healthy	CS	UBT	1, 2, 4, 5, 6	7
Assadi (11)	2009	Iran	53.20 ± 6.16	30/30	Community	healthy	CS	UBT	1, 2, 3, 4	7
Lanza (12)	2004	Italy	57 ± 8	55/60	Community	healthy	CS	Cag-A, UBT anti-HP/IgG	1, 2, 3, 4, 5, 6	6
Mehraban (13)	2012	Iran	53.8 ± 11.9	88/97	Community	healthy	CS	anti-HP/IgG	1, 2	7
Raeisi (14)	2012	Iran	51.8 ± 12.3	60/60	Community	healthy	CS	Cag-A, anti-HP/IgG	1, 2, 6	7
Seyyed-Mohammadzad (15)	2012	Iran	51.8 ± 12.3	100/100	Community	healthy	CS	Cag-A, anti-HP/IgG	1, 2, 6	7
Yu (16)	2018	China	51.2 ± 7.1	61/61	Hospital	healthy	CS	anti-HP/IgG	1, 2, 3, 45, 6	6
Song (17)	2007	China	45.41 ± 7.99	27/30	Hospital	healthy	CS	UBT	1, 2, 3, 4, 5, 6	7
Rasmi (18)	2016	Iran	53.8 ± 1.3	88/97	Hospital	healthy	CS	anti-HP/IgG	1, 2, 4	6
Li (19)	2018	China	60.1 ± 10.49	78/80	Hospital	healthy	CS	UBT	1, 2, 3, 4, 5, 6	7

*CSX, Cardiac syndrome X; CG, control group; CS, case-control study; anti-HP/IgG, anti-Helicobacter pylori /immunoglobulin G; UBT, urea breath test; Cag-A, cytotoxin-associated gene-A. Mehraban et al. study included Group A (Mehraban A: 25-40 years), Group B (Mehraban B: 40-55years) and Group C (Mehraban C: >55years). a Mean age = the mean age of the case group. b 1 = age, 2 = gender, 3 = smoking status, 4 = Blood lipids, 5 = Diabetes mellitus, 6 = Hypertension*.

### *Helicobacter pylori* Infection and Cardiac Syndrome X

**Figure 2:**
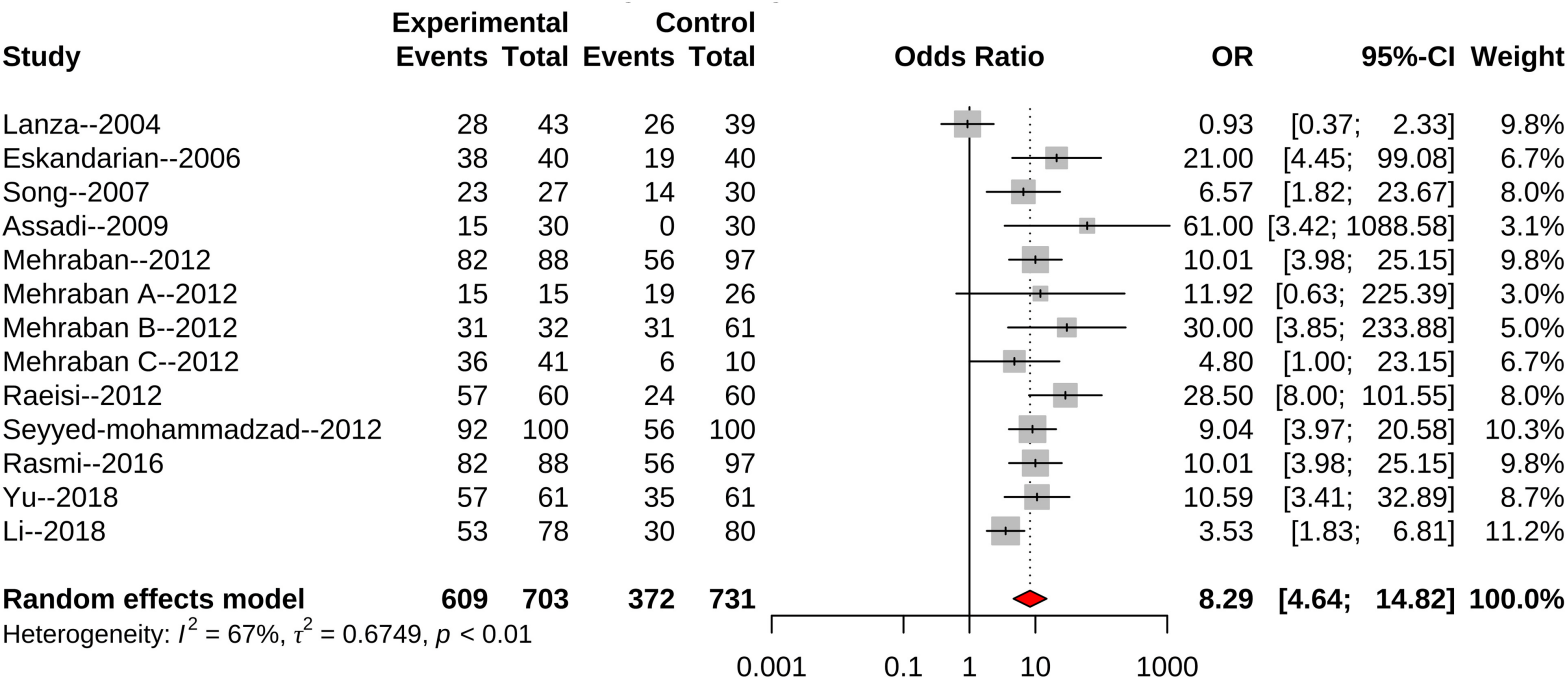
All identified articles evaluating *H. pylori* infection and the risk of CSX.

**Figure 3:**
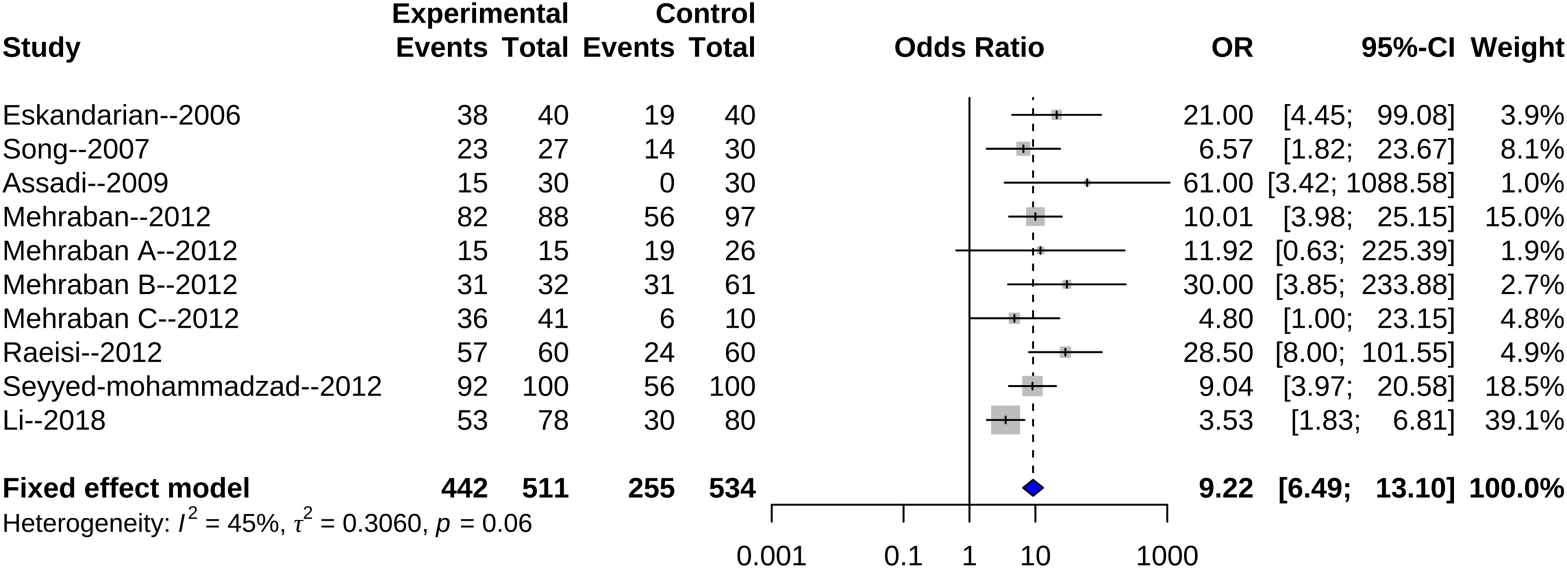
High quality articles evaluating *H. pylori* infection and the risk of CSX.

**Figure 4:**
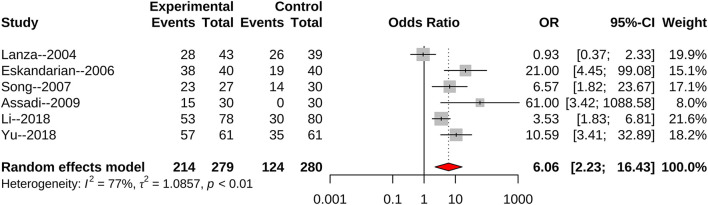
Articles with ≥4 matched variables to evaluate *H. pylori* infection and the risk of CSX.

We found 10 articles that reported an association of *H. pylori* infection with the risk of CSX. The combined random effect odds ratio (OR) was 8.29 (95% CI: 4.64–14.82) (Figure 2). After excluding three articles with low scores (six points), seven articles remained. Another meta-analysis showed that the combined fixed effect OR was 9.22 (95% CI: 6.49–13.10) (Figure 3). We also selected seven articles with ≥ four matched variables and performed meta-analysis. The combined random effect OR was 6.06 (95% CI: 2.23–16.43) (Figure 4).

The data were separately stratified by country, mean age, case type, *H. pylori* diagnostic methods, socioeconomic status and publication year. Two parallel meta-analyses were performed in each subgroup. Those results are presented in Table 2. Results suggested that the association of *H. pylori* infection with the risk of CSX may be much stronger in middle-aged individuals. Similarly, we found that this association is also more common in developing countries (China and Iran), but was not apparent in developed countries (Italy). When we excluded articles with six points, the meta-analysis was performed again. Differences in age and developing countries were still related to bacterial pathogenicity. Whether the articles with six points only or all included articles were analyzed, factors such as age or developing country were consistently observed to influence bacterial pathogenicity.

**Table 2:** Subgroup analyses about relationship between the risk of CSX and *H. pylori* infection.

**Subgroups**	**Number of studies^**a**^**	**OR(95% CI)**	***P* value for *I^**2**^***	**Number ** **of studies^**b**^**	**OR(95% CI)**	***P* value** **for *I^**2**^***
Country
Iran	6	12.99 (8.61–19.60)	0.59	5	13.84 (8.74–21.92)	0.50
China	3	5.14 (3.09–8.56)	0.23	2	4.05 (2.27–7.25)	0.40
Italy	1	0.93 (0.37–2.33)	NG	0	NG	NG
Age
25–40 years	1	1.34 (1.04–1.72)	NG	1	1.34 (1.04–1.72)	NG
40–50 years	2	11.27 (4.29–29.61)	NG	2	11.27 (4.29–29.64)	0.25
>50years	8	7.18 (3.59–14.36)	0.25	5	8.94 (4.38–20.58)	0.03
Case type
Community	6	8.34 (5.69–12.23)	<0.01	5	13.84 (8.74–21.92)	0.50
Hospital	4	6.16 (3.96–9.59)	<0.01	2	4.05 (2.27–7.25)	0.40
Diagnostic methods
UBT	5	5.46 (1.73–17.26)	0.19	4	8.69 (2.90–26.06)	0.05
anti-HP/IgG	6	8.26 (3.93–17.36)	<0.01	3	12.24 (7.49–20.01)	0.48
Socioeconomic status
Developed countries	1	0.93 (0.37–2.33)	NG	0	NG	NG
Developing countries	9	9.42 (6.87–12.91)	0.12	7	9.22 (6.49–13.10)	0.06
Publish year
<2010	4	7,41 (1.20–45.76)	<0.01	3	15.20 (6.19–37.32)	0.26
≥2010	6	8.74 (6.24–12.25)	0.12	4	8.96 (4.71–17.03)	0.05

*CSX, Cardiac syndrome X; NG, not given; anti-HP/IgG, anti-Helicobacter pylori/immunoglobulin G; UBT, urea breath test; a, all included articles; b, articles with six points*.

### CagA Strains and Cardiac Syndrome X

**Figure 5:**
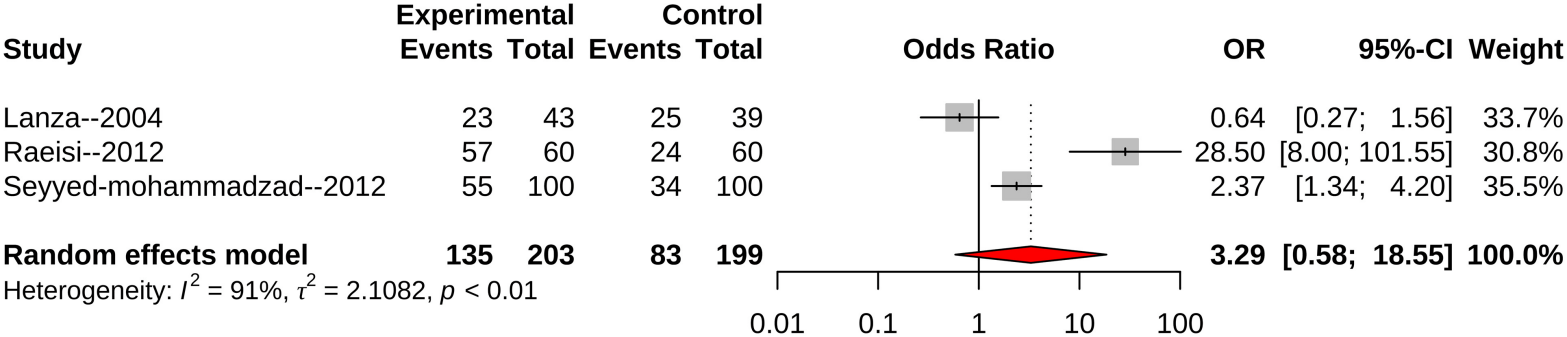
All identified articles evaluating CagA strain infection and the risk of CSX.

We also found three studies focusing on the association of CagA strain infection with the risk of CSX. The combined random effect OR was 3.29 (95%CI: 0.58–18.55 (Figure 5). Two studies scored seven points, with an OR 7.70 (95%CI: 0.65–91.37). Because of the lack of data, a subgroup meta-analysis of CagA strains could not be performed.

## Discussion

As far as we know, the relationship between *H. pylori* infection and the risk of CSX is still controversial, and our study is the first systematic review and meta-analysis on this topic. Based on the results of our meta-analysis, we estimate that the risk of CSX caused by the bacterial infection increases by ~87%.

**Figure 6:**
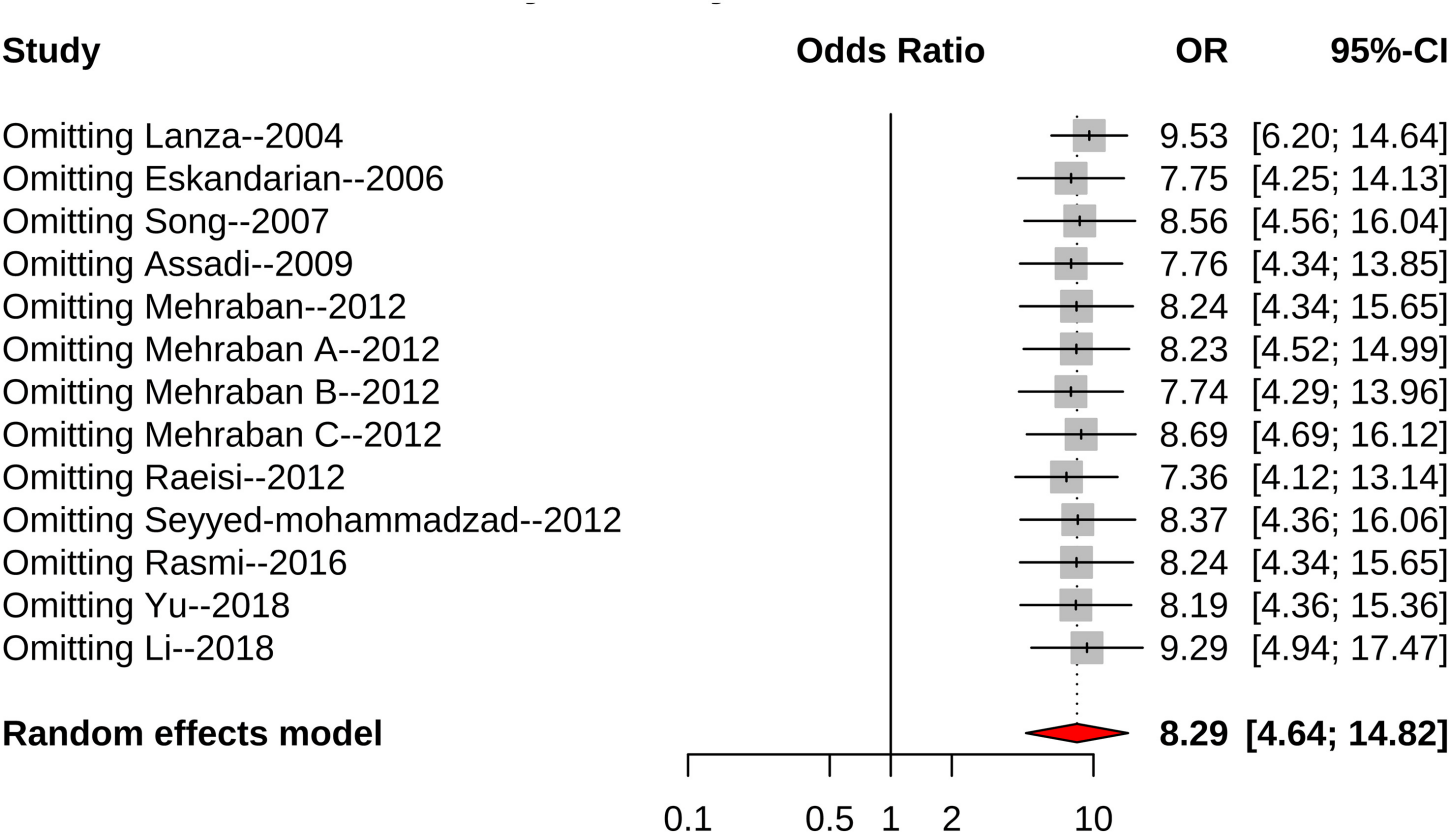
Forest plot for sensitivity analysis.

The meta-analysis included 10 articles, nine of which found that *H. pylori* infection increased the risk of CSX. However, one article held the opposite view. Each article in the meta-analysis had a weight range of 3.0–11.2%. Sensitivity analysis was further conducted and concluded that the deletion of any single article would not have a significant impact on the overall effect size (Figure 6). There was significant heterogeneity among included articles, which may be attributable to different study designs and different study populations.

In the meta-analysis, all articles achieved satisfactory scores (≥ six points) for quality assessment. Included articles with seven points yielded more reliable results, and there was no difference between studies with low scores (six points) and studies with high scores (seven points). We performed Egger's test (*P* > 0.05) and Begg's test (*P* > 0.05) and the results suggested no potential publication bias in our meta-analysis, assuring confidence in our results.

Several potential confounding factors, such as age, gender, and socioeconomic status, cannot be ignored because they are associated with *H pylori* infection and the risk of CSX. Age and gender were matched in all articles. A meta-analysis of articles with ≥ four matched variables was performed and the pooled result did not change. Thus, we believe that these confounding factors are evenly distributed in the control group and the case group. Potential confounding factors had no effect on the reliability of the results.

The meta-analysis showed that CagA-positive bacterial infections may not significantly increase the risk of CSX. However, we believe that this conclusion is controversial for two main reasons: first, CagA-positive strains showed high pathogenicity in a number of diseases including atherosclerotic diseases and peptic ulceration (20, 21). Second, the lack of data may be one reason for conflicting results as only three articles were included. As a result, more well-designed studies should be performed.

According to subgroup results, we found that the association between *H. pylori* infection and the risk of CSX was age-dependent, and the incidence rate of CSX was likely greater in middle-aged group than in other groups. This finding is similar to those of previous studies, which have shown that the association between the risk of CSX and *H. pylori* tends to be stronger in middle-aged people (16). We also found some preliminary evidence that the bacteria in developing countries are more pathogenic than in developed countries. Compared with developed countries, the annual recurrence rate of *H. pylori* in developing countries is higher, which may cause CXS to be more pathogenic (22). Additionally, although the *H. pylori*-immunoglobulin G (IgG) antibody test cannot indicate current infection and may overestimate the relationship between the bacteria and the risk of CSX, it is consistent with the results obtained by the urea breath test (UBT) test detects current infection.

After excluding low-scoring articles, several meta-analyses were performed, and differences in these subgroups still existed, supporting the reliability of our results. Previous studies have shown that the incidence rate of *H. pylori* infection was significantly increased with concurrent diseases, such as diabetes and malignant tumors (23, 24). The control group used in our study were all healthy individuals. Therefore, the selection of the control group fully takes into account the differences in *H. pylori* between the groups.

*H. pylori* infection is able to cause CSX through a variety of mechanisms. Chronic inflammation caused by infection enhances the risk of vascular disease by increasing some acute reactants and inflammatory mediators, leading to endothelial cell damage and blood coagulation (25, 26). Chronic infection caused by *H. pylori* infection, especially CagA-positive strains, can lead to continuously elevated inflammatory metabolites, such as the cytokines Interleukin-1 (IL-1), Interleukin-1 (IL-6), and tumor-necrotic factor (TNF-1), which can affect vascular activity and lead to endothelial dysfunction (5, 27). Also, Chronic *H. pylori* infection can cause a decrease in vitamin B12 and folate absorption, resulting in hyperhomocysteinemia, which promotes the production of intracellular oxygen free radicals and the degradation of nitric oxide, leading to endothelial cell dysfunction (16).

The strength of our study is that it is the first attempt using meta-analysis to identify the association of *H. pylori* infection with the risk of CSX. However, our study also has some limitations. First, due to lack of data, we mainly obtained the age and country information related to CSX individuals. Other factors we could not account for, such as gender and the instrument for measuring *H. pylori*, may also affect the accuracy of our results. Second, our study primarily focuses on three countries, which were divided into developed (Italy) and developing (China, Iran). An expansion of this analysis to other countries is needed to further verify our conclusions. Finally, the sample size of this meta-analysis is relatively small, which may affect the accuracy of our results. Therefore, additional, larger, well-designed studies should be encouraged to validate our results.

In conclusion, our meta-analysis suggested a possible association between *H. pylori* infection and the risk of CSX. Its pathogenicity is stronger in middle-aged individuals and some developing countries. However, more studies are needed to further investigate whether early eradication of *H. pylori* can reduce the incidence rate of CSX, especially in middle-aged individuals and some developing countries.

## Data Availability Statement

The original contributions presented in the study are included in the article/supplementary materials, further inquiries can be directed to the corresponding author/s.

## Author Contributions

D-HZ, KC, CY, and X-JD designed and analyzed the study. D-HZ, KC, CY, X-JD, and B-BW wrote and revised the manuscript. S-PL, F-HL, Z-XH, and X-LL collected the data. All authors have read and approved the manuscript.

## Conflict of Interest

The authors declare that the research was conducted in the absence of any commercial or financial relationships that could be construed as a potential conflict of interest.

## Publisher's Note

All claims expressed in this article are solely those of the authors and do not necessarily represent those of their affiliated organizations, or those of the publisher, the editors and the reviewers. Any product that may be evaluated in this article, or claim that may be made by its manufacturer, is not guaranteed or endorsed by the publisher.
